# Controlled dynamic screening of excitonic complexes in 2D semiconductors

**DOI:** 10.1038/s41598-017-18803-y

**Published:** 2018-01-15

**Authors:** Andrey R. Klots, Benjamin Weintrub, Dhiraj Prasai, Daniel Kidd, Kalman Varga, Kirill A. Velizhanin, Kirill I. Bolotin

**Affiliations:** 1 Department of Physics and Astronomy, Vanderbilt University, Nashville, TN-37235 USA; 2 Department of Physics, Freie University, Berlin, 14195 Germany; 3 Interdisciplinary Graduate Program in Materials Science, Vanderbilt University, Nashville, TN-37234 USA; 4 Theoretical Division, Los Alamos National Laboratory, Los Alamos, NM-87545 USA

## Abstract

We report a combined theoretical/experimental study of dynamic screening of excitons in media with frequency-dependent dielectric functions. We develop an analytical model showing that interparticle interactions in an exciton are screened in the range of frequencies from zero to the characteristic binding energy depending on the symmetries and transition energies of that exciton. The problem of the dynamic screening is then reduced to simply solving the Schrodinger equation with an *effectively frequency-independent* potential. Quantitative predictions of the model are experimentally verified using a test system: neutral, charged and defect-bound excitons in two-dimensional monolayer WS_2_, screened by metallic, liquid, and semiconducting environments. The screening-induced shifts of the excitonic peaks in photoluminescence spectra are in good agreement with our model.

## Introduction

Excitonic complexes (EC) including excitons, trions, and biexcitons are many-body bound states of electrons and holes that can be viewed as solid state analogs of atoms and molecules. Many fundamental atomic physics phenomena such as Bose-Einstein condensation, the Lamb shift, and the fine structure are also observed in ECs^1–3^. One of the key differences between ECs and atomic systems is the size – nanometers for ECs and Angstroms for atoms. While electric fields inside atoms are not perturbed by the environment, the fields in much larger ECs propagate into the surrounding medium and are screened by it. The dielectric properties of the environment can often be adequately described by a *dielectric constant*, *ε*. In that case, the EC binding energy, *E*_*bind*_, can be determined by solving the Schrodinger equation with screened interaction potential, *V*, calculated from the Poisson equation. Many realistic dielectrics, however, are characterized by a *dielectric function*, *ε*(*ω*), with pronounced frequency-dependence. In that much more complex but experimentally relevant case^4–6^, screening becomes *dynamic*, i.e. frequency-dependent. The following question arises naturally: how does one calculate the EC binding energies for frequency-dependent environments?

Effects of dynamic screening are especially interesting in two-dimensional semiconductors from the group of transition metal dichalcogenides (TMDCs). These materials feature a gamut of tightly-bound ECs with binding energies as large as 0.7eV^7,8^. The screening of the ECs, either by their microenvironment^5,9^ or by free carriers^10^, is especially strong due to the atomic thickness of TMDCs. So far, screening in TMDCs has been modeled as static with the dielectric constant taken either at zero^4,5^ or optical^4,11,12^ frequencies. While this approach is justified for some systems, for others it may lead to large errors. Although there have been no attempts – to the best of our knowledge – to examine dynamic screening of ECs in TMDCs, theoretical approaches have been developed for conventional semiconductors^13–16^. Unfortunately, these approaches rely on precise knowledge of properties of specific materials and/or require numerical solution of the Bethe-Salpeter equation, and hence are impractical for many realistic systems.

In this work, we develop an analytical model providing intuitive understanding of the screening process. Our model suggests that in order to obtain the energy levels of the dynamically screened ECs it is sufficient to consider the behavior of *ε*(*ω*) only within certain upper and lower frequency bounds, that in turn depend on internal properties of the EC. Further we show that even in the case of dynamic screening, EC binding energies can still be calculated using effectively static dielectric functions and screened interaction potentials evaluated at a certain fixed effective frequency that depends on EC symmetries. We experimentally test the model by studying ECs in monolayer TMDCs coupled to metallic, semiconducting, and liquid environments with frequency-dependent dielectric functions.

## Setting up the problem

The EC is a system of electrons (*e*) and holes (*h*) bound by an electric field, e.g. neutral exciton (*e* + *h*), charged exciton (2*e* + *h* or *e* + 2 *h*, also known as trion), defect-bound exciton (modeled as a trion with one particle being static), etc. We start with a simple semiclassical model of an exciton: two oppositely charged particles revolving around each other inside a homogeneous electrically polarizable medium. In the symmetric case of equally massive particles, *m*_*e*_ = *m*_*h*_, an electron and a hole revolve around their common center of mass with a frequency *ω*_*rot*_. The combined electric field of the particles and hence the polarization of the medium oscillate at the same frequency *ω*_*rot*_. In the opposite asymmetric case, *m*_*h*_ ≫ *m*_*e*_, the hole is static while the electron revolves around it. Correspondingly, the total electric field created by the charges will have both static and time-dependent components (see Supplementary Information S1). Thus, frequencies relevant for screening of interparticle interactions are expected to depend on EC symmetries in addition to the characteristic frequency *ω*_*rot*_ and related binding energy ${E}_{bind} \sim \hslash {\omega }_{rot}$.

We now approach the problem of dynamic screening analytically. Let EC eigenvectors, |*S*〉, and eigenenergies, *E*_*S*_, be the solutions of the *D*-dimensional Schrodinger equation with a frequency-independent interparticle interaction potential, *V*_0_. The screening becomes dynamic due to medium excitations, ${j}_{med}$, such as plasmons or phonons. The corresponding correction to the EC ground state energy can be obtained using the second-order perturbation theory:

1 $${\rm{\Delta }}{E}_{0}=-\sum _{S,j}\frac{{|\langle S|\langle {j}_{med}|{H}_{\mathrm{int}}|{0}_{med}\rangle |0\rangle |}^{2}}{{E}_{S0}+{E}_{j0}}.$$

Here, the perturbation ${H}_{int}=\int \rho (k){\rho }_{med}(-k){V}_{0}(k){d}^{D}k/{(2\pi )}^{D}$ describes Coulombic interactions between the EC and the medium, with ${\rho }_{(med)}(k)$ denoting exciton (medium) charge density in the momentum space. The summation is performed over all possible states of the EC and of the environment. The multi-index $S=\{n,q\}$ consists of an index *n* describing internal excitations of the EC (Rydberg series) and the total momentum *q* of the EC as a whole. Finally, ${E}_{S0}$ and ${E}_{j0}$ are the transition energies between ground and excited states of the EC and the medium respectively. Evidently, ${\rm{\Delta }}{E}_{0}$ depends on EC transition energies ${E}_{S0}$ starting with ${E}_{00}=0$. While exact expressions for $|{j}_{med}\rangle$ and ${H}_{int}$ depend on the structure of a particular solid state system and can be quite complex, their explicit forms are not required for calculating (1).

It is easy to see that the matrix element of the environment charge density entering (1) is directly related to the frequency-dependent environmental polarizability $\chi (q,\omega )$ written in the Lehmann representation^17^: ${|\langle {j}_{med}|{\rho }_{med}(q)|{0}_{med}\rangle |}^{2}\propto \text{Im}\chi (q,{E}_{j0})$. This relation allows us to express $|{j}_{med}\rangle$ and ${H}_{int}$, in terms of experimentally accessible dielectric functions of the medium. Then, the Poisson equation with medium dielectric constants evaluated at each frequency *ω* yields the dynamically screened *ω*-dependent interaction potential, $V(\omega )$. We note that $V(\omega )$ may have a complex spatial or, equivalently, momentum(*q*)*-*dependence. For example, in a two-dimensional material sandwiched between two dielectrics interparticle interactions are described by the Keldysh potential^18^. We, however, do not write this *q*-dependence explicitly, since our main focus is the frequency-dependence of interactions. The interaction potential $V(\omega )$ consists of an unperturbed frequency-independent potential *V*_0_ and a complex-valued dynamic term, ${V}_{s}(\omega )={V^{\prime} }_{s}(\omega )+i{V^{\prime\prime} }_{s}(\omega )$, henceforth referred to as the *screening potential*. Expressing the matrix elements of the perturbation ${H}_{int}$ via ${V}_{s}(\omega )$ we rewrite equation (1) without explicit involvement of ${j}_{med}$^13,14^:

2 $${\rm{\Delta }}{E}_{0}=-\frac{1}{2}\frac{1}{A}\sum _{S}{|{\rho }_{S0}|}^{2}{\mathop{V}\limits^{ \sim }}_{s}({E}_{S0}/\hslash ).$$

Here *A* is the crystal volume, ${\mathop{V}\limits^{ \sim }}_{s}({E}_{S0})=2{\pi }^{-1}{\int }_{0}^{\infty }{V^{\prime\prime} }_{s}(\omega ){(\omega +{E}_{S0}/\hslash )}^{-1}d\omega$^13^, and ${\rho }_{S0}=\langle S|\rho (q)|0\rangle$ is a charge density operator in momentum space “sandwiched” between EC ground and excited state-vectors. By analogy with transition dipole moment, ${\rho }_{S0}$ can be also called the transition charge density. Throughout the paper we use unitless elementary charge *e* = 1.

## Relevant screening frequencies

While it is possible to numerically compute ${\rm{\Delta }}{E}_{0}$ from equation (2), such calculations require evaluation of wavefunctions for all of the EC excited states. This is complex even for neutral excitons and impractical for larger ECs. However, we can further simplify equation (2) by using the general properties of ${\mathop{V}\limits^{ \sim }}_{s}$ and ${\rho }_{S0}$ (see Supplementary Information S1):

- The frequency-integral ${\tilde{V}}_{s}$ can be expressed, using the Kramers-Kronig relations, as frequency-smoothened real part of the screening potential, ${V^{\prime} }_{s}$:
  3 $${\mathop{V}\limits^{ \sim }}_{s}({E}_{S0})={\int }_{-\infty }^{\infty }f(\mathrm{ln}\,{E}_{S0}/\hslash -\,\mathrm{ln}\,\omega ){V^{\prime} }_{s}(\omega )d\,\mathrm{ln}\,\omega ,$$
  where $f(x)=2{\pi }^{-2}x/\,\sinh \,x$ is a normalized bell-shaped distribution function with a vanishing mean value and standard deviation of ~2. According to (3), ${\mathop{V}\limits^{ \sim }}_{s}({E}_{S0})$ can simply be approximated by a real part of the screening potential ${\mathop{V}\limits^{ \sim }}_{s}({E}_{S0})\cong {V^{\prime} }_{s}({E}_{S0}/\hslash )$, provided that ${V^{\prime} }_{s}(\omega )$ is a slow-varying function of frequency. This approximation is valid for many real media^19–22^ and is used henceforth to simplify derivations. Furthermore, such frequency-smoothened potential turns out to be free of sharp irregularities caused by lattice excitations. For example, if a potential screened by a Lorentz medium with a divergence at a frequency *ω*_0_ (typically in the mid-IR range) has a shape of ${V}_{s}(\omega )\propto {({\omega }^{2}-{\omega }_{0}^{2})}^{-1}$, then the transformed potential ${\mathop{V}\limits^{ \sim }}_{s}({E}_{S0})\propto {({E}_{S0}/\hslash +{\omega }_{0})}^{-1}$ is always smooth since ${E}_{S0}\ge 0$. This means that lattice excitations of the medium and other spectral irregularities or “kinks” do not have a strong effect on the dynamic screening of ECs.
- The transition charge density created by an electron and a hole vanishes – as can be shown analytically – if |0〉 and |*S*〉 are both symmetric with respect to exchange between electron and hole coordinates ${r}_{e}\leftrightarrow {r}_{h}$. In the case of such *symmetric transition*, the contributions to ${\rho }_{S0}$ from an electron and a hole are equal in magnitude and opposite in sign and therefore cancel each other out. Thus, only the *asymmetric* transitions contribute to the sum in (2). This condition is analogous to selection rules in atomic physics. As a result, the minimal value, ${E}_{\min }$, of the transition energy ${E}_{S0}$ contributing to the sum in (2) is the *energy difference* between *the ground state* and *the lowest asymmetric state*. The summation in equations (1 and 2) also has a characteristic upper-bound cutoff energy of the order of the EC binding energy, ${E}_{\max } \sim |{E}_{bind}|$^23,24^: due to decreasing overlap between $|0\rangle$ and $|S\rangle$, the terms corresponding to transition energies above that cutoff quickly decay with increasing ${E}_{S0}$, allowing the sum in (1, 2) to converge. Thus, only some of the lower-energy terms in (2) effectively contribute to ${\rm{\Delta }}{E}_{0}$. This means that in order to investigate dynamic screening of the ECs one needs to consider the behavior of the dielectric functions only within a certain frequency range between ${E}_{\min }$ and ${E}_{\max }$.
- The summation in equation (2) can be further simplified by replacing the frequency-dependent function ${V^{\prime} }_{s}({E}_{S0}/\hslash )$ by a frequency-independent mean value ${V^{\prime} }_{s}({E}_{eff}/\hslash )$ where the effective energy, ${E}_{eff}$, is a constant lying between the lower and upper energy bounds, ${E}_{\min } < {E}_{eff} < {E}_{\max }$. This assumption of effectively static screening allows one to treat the EC as a set of particles interacting via frequency-independent potential ${V}_{0}+{V^{\prime} }_{s}({E}_{eff}/\hslash )=\mathrm{Re}V({E}_{eff}/\hslash )$. In this case, the perturbed ground state energy is

4 $${E}_{0}+{\rm{\Delta }}{E}_{0}=\langle 0|T+\frac{1}{2}\sum _{j,k}{Q}_{j}{Q}_{k}({V}_{0}({r}_{jk})+{V^{\prime} }_{s}({r}_{jk},{E}_{eff}/\hslash ))|0\rangle ,$$

where *Q*_*j*_ is the charge of the *j*-th particle, ${r}_{jk}$ is the interparticle distance and *T* is the total kinetic energy of all the particles in the EC.

It is instructive to consider examples clarifying the evaluation of the lower-bound energy *E*_min_. In the case of a neutral exciton with equal electron and hole masses^25^, the ground state *n* = 0 is symmetric. For a realistic system of *nearly equal e*- and *h*-masses in TMDC, ${\rho }_{00}$ is proportional to the mass discrepancy between an electron and a hole (2 ~ 20%)^25^. Hence, $|{\rho }_{00}{|}^{2}$ entering (2) does not exceed ~4% compared to the case of unequal e/h-masses. Then, the energy of the first *asymmetric* transition is ${E}_{\min }\approx {E}_{1,0}={E}_{n=1}-{E}_{n=0}$, which typically is of the same order as $|{E}_{bind}|$^4^. Other common ECs such as trions, defect-bound excitons or neutral excitons with uneven *e*- and *h*-masses behave differently. Their ground state wavefunctions are inherently asymmetric with respect to ${r}_{e}\leftrightarrow {r}_{h}$ exchange^7^. The lowest asymmetric transition for such ECs is purely translational (with no change in *n*) with ${E}_{\min }\to 0$. Realistically, an EC may decay before the medium has enough time to get fully polarized. Hence, the effective ${E}_{\min }$ is not exactly zero, but is limited by the inverse characteristic lifetime $\sim {\tau }^{-1}$ of the particles constituting the EC.

Equations (3, 4) along with the estimates of ${E}_{eff}$ constitute our main theoretical result. In (4), we effectively replace the dynamically screening medium by a medium with a static dielectric constant $\varepsilon ({E}_{eff}/\hslash )$. To enable experimental predictions from (4), we note that the ‘diagonal’ terms with $k=j$ represent *self-interaction* of each carrier with its image charges. ‘Off-diagonal’ terms with $k\ne j$ account for screening of *interparticle interactions* (i.e. EC binding). Within simple, but widely used effective-medium approximations for interaction potentials, the calculation of self-energies is very susceptible to small uncertainties in microscopic structure of the investigated system and can even yield divergent results^24^. However, the *effective binding energy*, calculated using only off-diagonal ($k\ne j$) terms in (4), can still serve as a proxy for evaluating strength of interparticle interactions, screened by the medium with *effective* dielectric constant $\varepsilon ({E}_{eff}/\hslash )$.

In summary: the range of binding energies of ECs *dynamically* screened by environment with dielectric function $\varepsilon (\omega )$ can be evaluated, to the second order of the perturbation theory, by simply solving the EC Schrodinger equation with the *effective dielectric constants*, obtained from the true frequency-dependent dielectric function evaluated at two limiting frequencies: ${\omega }_{\min }={E}_{\min }/\hslash$ and ${\omega }_{\max }={E}_{\max }/\hslash  \sim |{E}_{bind}|/\hslash$. Binding energies obtained from these two cases are the upper and the lower bounds for the actual EC binding energy. The lower bound depends on the EC symmetry: ${E}_{\min }\approx {E}_{1,0} \sim |{E}_{bind}|$ for symmetric charge-neutral ECs with equal *e/h* masses and ${E}_{\min } \sim \hslash /\tau$ (inverse lifetime of particles constituting the EC) for asymmetric ECs with unequal *e*/*h*-masses or non-zero net charge. In some specific cases the problem can be simplified further. For example, in the case of a long-lived exciton with *m*_*h*_ ≫ *m*_*e*_, a heavy hole can be effectively treated as static and its field – as constant. Such a field, and hence, exciton binding will be screened by the medium only at zero effective frequency $\omega =0$ yielding a static effective dielectric constant $\varepsilon (\omega =0)$. Below we will demonstrate that for many realistic cases, *ε* does not change significantly between frequencies ${E}_{\min }/\hslash$ and ${E}_{\max }/\hslash$. This allows us to make experimentally testable predictions regarding screening of EC binding despite the simplicity and generality of the developed approach. Although the developed approach deals with the frequency and energy ranges rather than with precise numerical values, in the following section we will demonstrate that it allows to make experimentally testable predictions regarding the dynamic screening of ECs. These predictions follow from simple equations (3) and (4) and can be carried out with minimal computational resources.

## Setting up the experiment

**Figure 1:**
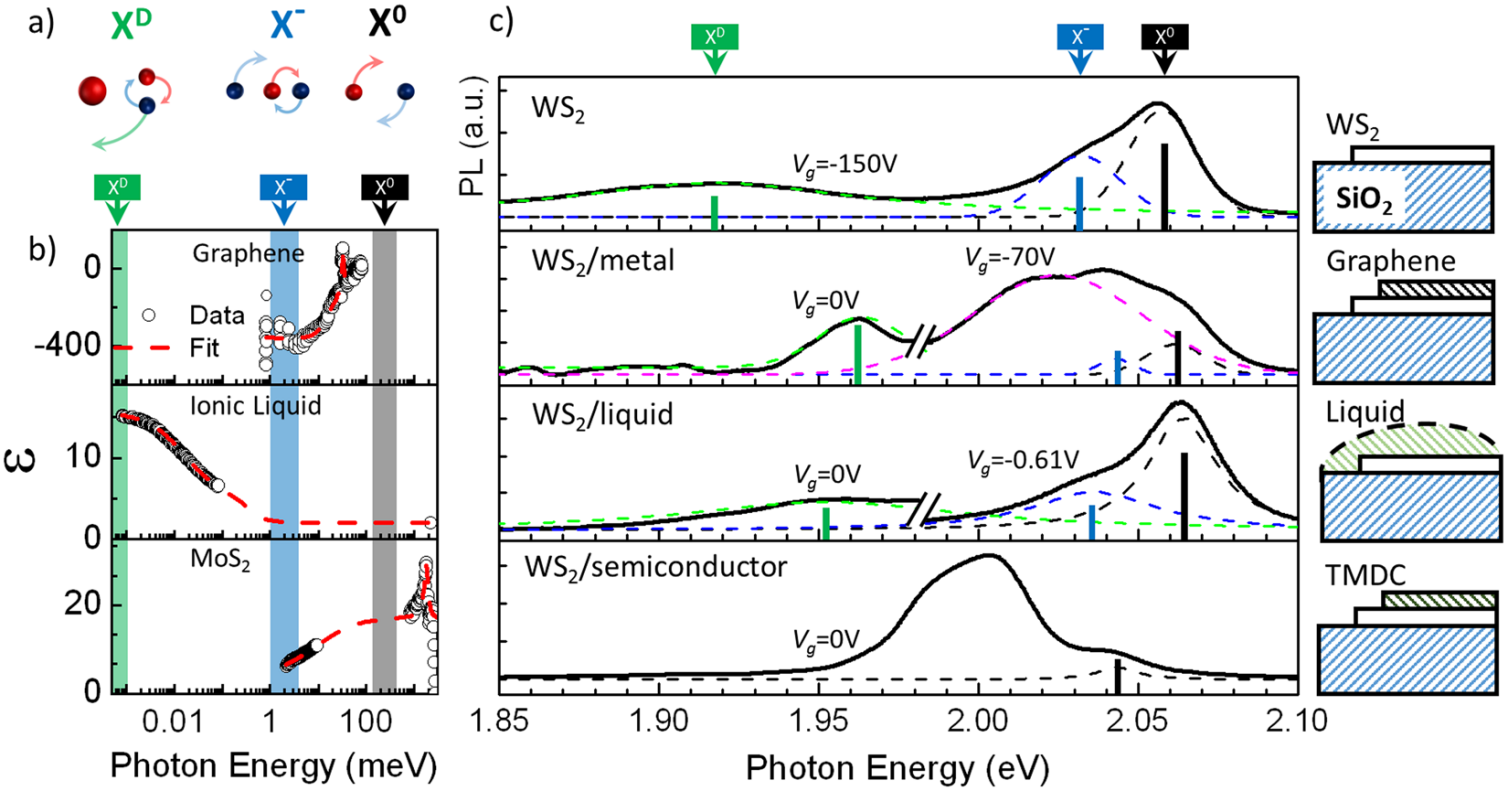
Effect of environments on WS_2_ PL spectra. **(a)** top: schematic illustrations of X^D^ (static impurity is in the middle), X^−^ and X^0^. **(b)** Dielectric functions of the screening materials: graphene^19^, ionic liquid^20,21^, and monolayer MoS_2_^22^. Since experimental dielectric functions are not available for the entire frequency range, we interpolate them using double Lorentzian fitting. Although it is possible that sharp features arising from the lattice excitations may exists between the experimentally verified data points, they – as shown above – do not contribute to ${\mathop{V}\limits^{ \sim }}_{s}$. **(c)** PL spectra of WS_2_ in different environments – schematics are on the right. Dashed curves are fitted excitonic peaks. The symbol “//” separates curves obtained from different samples/at different gate voltages. Gate voltage at which the curve was recorded is shown above each curve. As *in-situ* gating with ionic liquid is impossible at low temperatures, the data for the WS_2_/liquid device (right curve) were obtained at 240 K and artificially blue-shifted by 40 meV to account for thermal shift of the peaks^27^.

In order to test the developed theory, we measure the effect of different dispersive environments on binding energies of different types of ECs in a monolayer TMDC. We choose monolayer WS_2_ as a test bed since this material has a variety of tightly bound ECs^4,8,10,26–28^ that produce narrow and well-resolved peaks in photoluminescence (PL) spectra^4,8,10,27,29^. Note that in the tungsten-based materials, excitons experience a spin-splitting of ~11 meV ^30^. Due to the optical selection rules, only one higher-energy state is optically bright and can be observed experimentally^31^. We focus on three prominent excitonic species (Fig. 1a):

- Neutral exciton (X°). It has nearly identical electron and hole masses^7,25^ and is symmetric according to our classification. Therefore, interparticle interactions are expected to be screened at an effective energy in the mid-IR range: between the first excited state transition energy of ~130 meV^4^ and binding energy of ~320 meV^4^.
- Trion (X^−^). This charged state is classified as asymmetric. In the case of trion, we expect screening in the THz range: between ~0.5 meV, which corresponds to ~10 ps lifetime^32,33^, and the binding energy ~30 meV^8^.
- Defect-bound exciton^26,28^ (X^D^), treated here as a neutral exciton bound to a static charged impurity. Although currently the origin of impurities is not completely clear, the observed ~150 meV binding energy of X^D^ agrees with our numerical model (*e* + *h* + static charge) described below. Note that the binding energies of X^D^ and X^−^ are defined with respect to the energy of a neutral exciton. The electric field of a static charged impurity, binding the exciton, is screened at zero frequency. Since it is energetically favorable to have an electron (hole) highly localized near a static impurity and hole (electron) - delocalized, the corresponding distribution of the density function makes the defect-bound exciton similar to a highly asymmetric neutral exciton described above. Thus, defect-bound excitons are expected to be screened at zero frequency.

To test the dynamic screening of these ECs, we choose the media with qualitatively different dielectric functions in the range of relevant frequencies (Fig. 1b):

(i) Metallic medium. Two-dimensional semimetal graphene exemplifies a metallic-type dielectric response *ε ∝ω*^−2^. Specifically, *ε*(*ω*) for graphene is large (>10) for *ω* from 0 to THz and is close to 1 in the IR range.
(ii) Liquid medium. We use the ionic liquid *Diethylmethyl(2-methoxyethyl)ammonium bis(trifluoromethylsulfonyl)imide*, for which *ε*(*ω*) is large (>10) at sub-GHz frequencies and is insignificant above 1 THz.
(iii) Semiconducting medium. For semiconductors, *ε*(*ω*) is roughly constant in a broad range of frequencies. In our experiments, monolayer MoS_2_ transferred onto our device serves as a semiconducting screening layer with *ε*(*ω*)~15 in IR-to-visible range and ~5 in the sub-THz range.

Figure 1b shows the dielectric functions for each medium along with the frequency ranges (shown as vertical bands) relevant for screening of X°, X^−^, and X^D^. The dielectric functions are relatively constant within each band. Summarizing, we expect the binding energy of neutral excitons to be strongly affected by semiconducting but not liquid or metallic environments. For trions, we expect strong screening by metallic environment only. Finally, defect-bound excitons should be affected by metallic and liquid environments. We cannot make a definitive qualitative prediction of the effect of the semiconducting medium on X^−^ and X^D^ because, in relevant sub-THz range, MoS_2_ dielectric constant (*ε*~5) is neither large (>10) nor small (~1).

## Measurements

Measurements were performed on monolayer WS_2_ flakes exfoliated on Si/SiO_2_ substrates. Electrostatic gating was used to control the Fermi level and isolate the contribution of free-carrier screening^8,10^. In order to study X^D^, we induced defects using argon plasma^26^. We begin our measurements by recording PL spectra (532 nm, ~20 µW laser excitation focused into a ~2 µm spot) at *T* = 78 K for pristine WS_2_ devices without any material on top (Fig. 1c, WS_2_ device). The well-known peaks in the PL spectra at ~2.06 eV (black dashed line), ~2.03 eV (blue dashed line), ~1.92 eV (green dashed line) are identified as stemming from neutral excitons X^0^, trions X^−^ and defect-bound excitons X^D^ respectively^7,8,10,26,27^. The peak at ~2.02 eV observed in some devices (e.g. Fig. 1c, pink dashed line) is likely associated with an additional trion state^2,10,34^ and is not analyzed further.

We modify the dielectric environment of the WS_2_ flake by either mechanically transferring^35^ monolayer graphene or MoS_2_ (WS_2_/metal and WS_2_/semiconductor device respectively), or dropcasting a layer of ionic liquid (WS_2_/liquid device). We then re-acquire the PL spectra. We observe large and reproducible shifts of all three excitonic peaks (Fig. 1c). Note that environmental factors other than screening (i.e. induced doping, strain, and chemical modifications) may also cause peak shifts^8,10,36,37^. However, as shown below and in Supplementary Information S3, the observed shifts are too strong to be explained by changes in the doping level. The effects of strain are shown to be weak by comparing PL spectra of transferred heterostructures and naturally grown WS_2_ bilayers. We also see no evidence of chemical modifications in WS_2_/liquid devices as observed shifts are reversed by *removing* the ionic liquid. Thus, we interpret observed shifts as originating from the dielectric screening of excitons. To compare these shifts with theory, we extract exciton binding energies for different types of environment. The binding energies of trions and defect-bound excitons are determined as $|{E}_{bind}({{\rm{X}}}^{-,D})|=Pos({{\rm{X}}}^{-,D})-Pos({{\rm{X}}}^{0})$, where *Pos*(*X*) is the energy position of a particular excitonic peak in the PL spectrum. In pristine devices, we observe $|{E}_{bind}({{\rm{X}}}^{-})|$ ~ 25 meV and $|{E}_{bind}({{\rm{X}}}^{D})|$ ~ 140 meV, close to literature values^8,10,26^.

Unfortunately, $|{E}_{bind}({{\rm{X}}}^{0})|$ cannot be measured directly using absorption or PL spectroscopies as these techniques are unable to directly probe the single-particle electronic bandgap^4,8,38^. We rely on the on the experiments by Chernikov *et al*.^4,10^ measuring $|{E}_{bind}({{\rm{X}}}^{0})|$ ~ 320 meV for uncovered Si/SiO_2_/WS_2_ devices similar to ours, and showing 1 meV red-shift in *Pos*(*X*^0^) per ~6 meV decrease in the exciton binding energy (studied by controlling the interparticle interactions by either varying the number of layers or the carrier density in WS_2_). These observations allow us to convert the screening-induced shifts of the X^0^ PL peak position into its effective binding energy.

**Figure 2:**
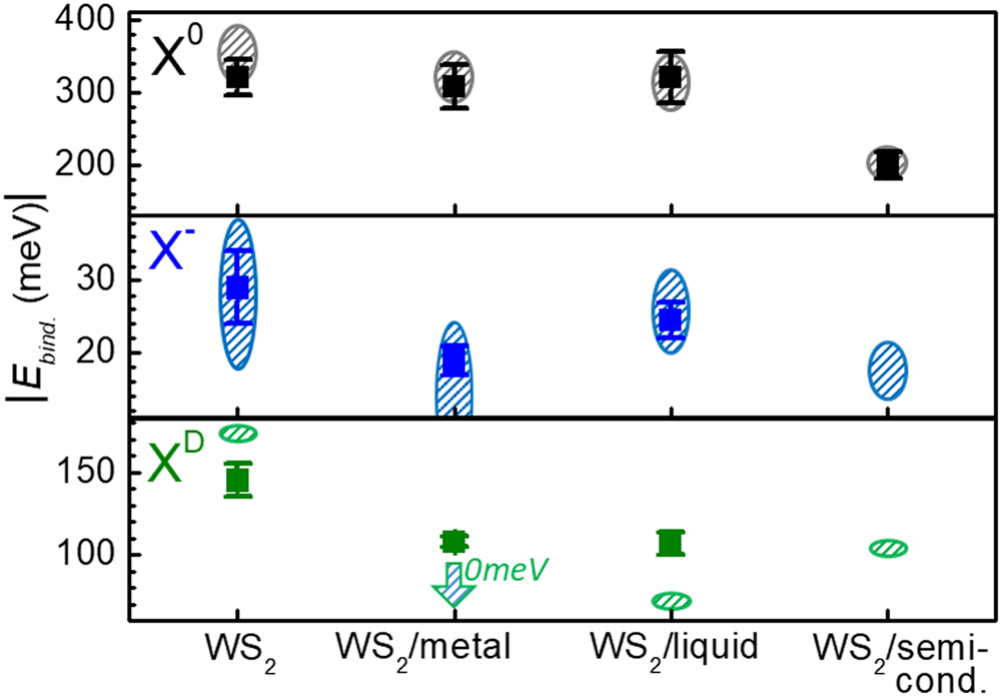
Summary of experimental and theoretical results. Square symbols are experimentally observed EC binding energies in presence of different screening materials, while ovals show the range of theoretically predicted values. For both X- and X^D^ in WS^2^/metal devices the calculated energy range starts at zero (shown by downward arrow in the case of X^D^).

Figure 2 summarizing the effects of metallic, semiconducting, and liquid environments on the binding energies of X^0^, X^−^, and X^D^ (square symbols) constitutes our main experimental result. The following trends are evident: The extracted binding energy of X^0^ decreases by 120 ± 40 meV (~40%) in the WS_2_/semiconductor sample. This conforms well with studies performed on bi- and multi-layer TMDCs^4,39,40^. For X^−^, the binding energy is downshifted by 10 ± 3 meV (~30%) due to the presence of graphene. The binding energy of X^D^ is reduced by 40 ± 20 meV (~30%) in presence of both metallic and liquid environments. In all other measured cases EC peak shifts are insignificant within our error bars. These trends agree well with our qualitative predictions. In the case of WS_2_/metal and WS_2_/semiconductor samples we could not bring WS_2_ close to depletion, likely due to strong effects of charge transfer in these heterostructures^41^.

Nevertheless, observed shifts exceed possible doping-induced effects: the trion binding energy in presence of graphene becomes as low as 19 meV, and the neutral exciton red-shifts to 2.045 eV in semiconductor-capped devices. These values are significantly below the energies achieved by doping alone^8,10^ (see Supplementary Information S3).

## Quantitative comparison with theory

To further verify our model, we perform quantitative estimates of screening-induced changes in ECs energies (see Supplementary Information S2). We computationally solve the Schrodinger equation for 2- or 3-body systems using a variational approach^42–44^ with *e*- and *h*-masses of 0.45 *m*_0_^25,45^ and infinite mass for the defect charge. Interparticle interactions are modelled by the Keldysh potential^18^ calculated using effective WS_2_ and medium dielectric functions taken at effective frequency *ω*. Upper- and lower-bound estimates for EC effective binding energies (${E}_{bind}({\omega }_{\min })$ and ${E}_{bind}({\omega }_{\max })$) are obtained by setting *ω* to ${\omega }_{\min }={E}_{\min }/\hslash$ or ${\omega }_{\max }=|{E}_{bind}|/\hslash$ as prescribed by our theoretical model. We assume that the dielectric functions of all the materials depend on frequencies but not on wavenumbers. This is because the characteristic spatial dimension of excitons in TMDCs is significantly greater compared to the lattice constant but significantly smaller than the mean distance between charge carriers (see Supplementary Information S3.2). The values ${E}_{bind}({\omega }_{\min /\max })$, acting as proxies for expected shifts of EC energy levels, can now be compared to experimentally observed values.

The ranges of calculated effective EC binding energies – from ${E}_{bind}({\omega }_{\min })$ to ${E}_{bind}({\omega }_{\max })$ – are shown as shaded ovals in Fig. 2. Our computational results are in agreement with values obtained via different methods by other groups^46,47^. Experimentally observed values of X^0^ and X^−^ binding energies are within the theoretically expected range for all media. Shifts of X^D^, calculated assuming only zero-frequency screening, exceed experimental ones, probably due finite spatial separation between the measured EC and the medium, which is assumed to be negligible in our model. In the case of X^−^ and X^D^ in the presence of a semiconductor environment, predicted shifts are too subtle to be experimentally tested with certainty and were not measured as that would require higher accuracy of computational models and measurement techniques. Overall, we believe that this quantitative agreement is remarkable for a minimal model with no free parameters.

## Conclusions

The theory of excitonic complexes in dynamically-screening media was developed and confirmed experimentally. We obtained the binding energies of dynamically screened ECs by solving the Schrodinger equation with *effectively static* interaction potentials calculated at the *fixed effective* frequency. This frequency depends on the symmetries of the wavefunctions and the binding energies of ECs. The model was tested and confirmed experimentally by using neutral, charged, and defect-bound excitons in monolayer WS_2_ screened by metallic, semiconducting, and liquid environments. The developed approach is general and can be applied to diverse systems of quasiparticles, interacting via electric fields: including plasmons, excitonic molecules, and polaritons, screened by various media.

Our simple dynamic screening model may help to re-interpret and clarify a wide range of previous experiments where static screening was assumed. For example, the assumption of zero-frequency screening of two-dimensional ECs by liquids ($\varepsilon (\omega =0) \sim 50$) has led to the appearance of outlying data points, overestimation of exciton binding energies^5,48^ and underestimation of the effective electron mass by two orders of magnitude^49^. Moderate shifts in exciton energies observed in these experiments are more consistent with screening at optical frequencies, as predicted by our model, where most liquids have *ε* ~ 2. Another important example is the inconsistency in the reported neutral exciton binding energy in monolayer MoS_2_, which ranges from 220 meV to 660 meV depending on the type of measurements and applied models^38,50,51^. The lowest binding energy, 220 meV, was obtained by Zhang et *al*.^51^ by subtracting the optically measured energy of the excitonic PL peak from the electronic bandgap measured using scanning tunneling spectroscopy. Their measurements were performed using MoS_2_ samples on a semimetallic graphite substrate. According to our model, excitonic and free-particle states are screened by graphite at different effective frequencies, which yields ~400 meV difference in corresponding screening-induced energy shifts. This accounts for the discrepancy between the values obtained by Zhang *et al*. and by others^38,50^.

Effects of dynamic screening may also have practical applications. For example, it may be possible to probe frequency-dependent dielectric functions of various microscopic environments by measuring relative shifts of different types of ECs (including EC excited states) that are screened at different effective frequencies. This can be interesting for label-free biodetection or chemical sensing.

## Data Availability

The data generated or analyzed during this study are included in this published article (and its Supplementary Information files). All additional datasets generated during and/or analyzed during the current study are available from the corresponding author on reasonable request.

## Electronic supplementary material

Supplementary Information
