# Publisher Correction: Controlled dynamic screening of excitonic complexes in 2D semiconductors

**DOI:** 10.1038/s41598-018-23600-2

**Published:** 2018-04-12

**Authors:** Andrey R. Klots, Benjamin Weintrub, Dhiraj Prasai, Daniel Kidd, Kalman Varga, Kirill A. Velizhanin, Kirill I. Bolotin

**Affiliations:** 1 Department of Physics and Astronomy, Vanderbilt University, Nashville, TN-37235 USA; 2 Department of Physics, Freie University, Berlin, 14195 Germany; 3 Interdisciplinary Graduate Program in Materials Science, Vanderbilt University, Nashville, TN-37234 USA; 4 Theoretical Division, Los Alamos National Laboratory, Los Alamos, NM-87545 USA

Correction to: *Scientific Reports* 10.1038/s41598-017-18803-y, published online 15 January 2018

In the original version of this Article, Figure 2 was omitted.

Additionally, in the section ‘Setting up the experiment’,

“*Dirthyl methyl (2-methoxyethyl) ammonium bis (trifluoromethylsulfonyl) imide*”

now reads:

“*Diethylmethyl(2-methoxyethyl)ammonium bis(trifluoromethylsulfonyl)imide*”

Finally, the sections “Setting up the experiment”, “Measurements”, “ Quantitative comparisons with theory”, “Conclusions”, and “Data Availability” were incorrectly listed as subheadings in the original version of the Article.

These errors have now been corrected in the PDF and HTML versions of the Article.

