# Supplementary material for: Controlled dynamic screening of excitonic complexes in 2D semiconductors
Source: Sci Rep. 2018 Jan 15;8:768. doi: 10.1038/s41598-017-18803-y (PMC5768700; doi:10.1038/s41598-017-18803-y)
Supplement: Supplementary file 1 — Supplementary Information [file 41598_2017_18803_MOESM1_ESM.pdf]

Controlled dynamic screening of excitonic complexes in 2D semiconductors

Andrey R. Klots[1], Benjamin Weintrub[1,2], Dhiraj Prasai[3], Daniel Kidd[1], Kalman Varga[1], Kirill A. Velizhanin[4], Kirill I. Bolotin[1,2]

[1]Department of Physics and Astronomy, Vanderbilt University, Nashville, TN-37235, USA

[2]Department of Physics, Freie Univerity, Berlin-14195, Germany

[3]Interdisciplinary Graduate Program in Materials Science, Vanderbilt University, Nashville, TN-37234, USA

[4]Theoretical Division, Los Alamos National Laboratory, Los Alamos, NM-87545, USA

Supplementary Information

S1. Theoretical approach.......2

S1.1. Semiclassical model.......2

S1.2 Charge density matrix element in center-of-mass frame.......2

S1.3. Simplified energy correction.......4

S1.3. Selection rule for transition charge densities.......4

S1.4. Charge density matrix element for unbound states.......5

S1.5. Integral of imaginary part of potential. Its physical significance and properties.......6

S1.6. Approximation for dynamic screening.......7

S2. Modelling of ECs in 2D systems.......9

S2.1. Analysis of interaction potential.......9

S2.2. Numerical calculations.......11

S3. Artifact analysis.......13

S3.1. WS_2 devices in liquids. Chemical modification.......13

S3.2. Screening by free carriers.......14

S3.3. Effects of strain.......15

S1. Theoretical approach.

S1.1. Semiclassical model.

Let us consider dielectric screening of the electric field in a classical model of an EC: two charged particles rotating around each other in a dielectric medium. We assume that the particles are rotating with the frequency ω and are separated by the distance ρ. We first consider the symmetric case of equally massive and oppositely charged (with charge Q) particles rotating around the common center of mass. The displacement field D at any point of space r can be expressed as the gradient of the potential created by the particles:

$$D=-\nabla\frac{Q}{\sqrt{|r|^2+|\rho|^2/4}}\left[\frac{1}{\sqrt{1+\frac{|r||\rho|}{|r|^2+|\rho|^2/4}\cos\omega t}}-\frac{1}{\sqrt{1-\frac{|r||\rho|}{|r|^2+|\rho|^2/4}\cos\omega t}}\right]$$

In this case, the displacement field is clearly the odd function of $\cos\omega t$. Hence, D has only odd harmonics with frequencies $(2n+1)\omega$ (here $n\in Z$) and hence, will not have any zero-frequency harmonics.

In the opposite asymmetric case of much heavier positive (for instance) particle, that particle is considered static. The displacement field reads:

$$D=-\nabla\left[\frac{Q}{\sqrt{|r|^2}}-\frac{Q}{\sqrt{|r|^2+|\rho|^2-2|r||\rho|\cos\omega t}}\right].$$

This expression for displacement field is neither symmetric nor antisymmetric function of $\cos\omega t$. This function has all of the harmonics with frequencies $2n\omega$, including the zero-frequency mode. Therefore, the contribution of the dielectric response of the medium at zero frequency to screening depends on the symmetry of the EC.

S1.2 Charge density matrix element in the center-of-mass frame.

In this section, we derive the expression for the charge density matrix element and derive equation (2) of the main text. We start with expression for the screening-induced perturbation correction of the ground state energy of the excitonic complex (EC) in the second order of the perturbation theory[1,2]:

$$\Delta E_0=-\frac{1}{2}\frac{1}{A}\sum_{S,q}|\rho_{0S}(q)|^2\frac{2}{\pi}\int_0^\infty\frac{\mathrm{Im}V_s(\omega,q)}{\omega+E_{S0}}d\omega. \qquad \text{(S1.2.1)}$$

Here and further in the text we use atomic units $e=\hbar=1$. As mentioned in the main text, multi-index $S=\{n,k\}$ consists of n - the index labelling internal excitations of the EC in the center-of-mass frame and k - momentum of the EC as a whole. Fourier-transformed screening potential $V_s(\omega,q)$ depends on wavenumber q and frequency ω. To simplify (S1.2.1), we perform the calculation in the center-of-mass frame. We write the wavefunction of EC with momentum k and internal quantum number n as

$$\psi_S\equiv\psi_{k,n}=A^{-1/2}\exp(ikr_C)\Psi_n(r_1',r_2',...), \qquad \text{(S1.2.2)}$$

where r_C is the coordinate of EC's center of mass and Ψ_n is the center-of-mass wavefunction. Coordinate r'_j is a center-of-mass (CM) frame coordinate (denoted by the "prime"-symbol) of *j*-th particle. Coordinates in a static/laboratory reference frame will be denoted as $r_1, r_2, ...$ (no "prime"). Charge density of an *N*-particle excitonic complex is written as $\rho(r) = \sum_{j=1}^{N} Q_j \delta(r - r_j)$, where Q_j is the charge of j-th particle. Fourier transform of $\rho(r)$ yields $\rho(q) = \sum_{j=1}^{N} Q_j \exp(-iqr_j)$. Sandwiching $\rho(q)$ between vectors $\langle 0| = \langle 0,0|$ and $|S\rangle = |n,k\rangle$, we get:

$$\rho_{0S}(q) = \sum_{j=1}^{N} Q_j \langle 0| e^{-iqr_j} |S\rangle \equiv \sum_{j=1}^{N} Q_j \rho_{0S}^j \quad . \tag{S1.2.3}$$

Each term in (S1.2.3) can be written as:

$$\langle 0| e^{-iqr_j} |S\rangle = \langle 0,0| e^{-iqr_j} |n,k\rangle = A^{-1} \int \Psi_0^* \Psi_n e^{ikr_C} e^{-iqr_j} \delta\left(\sum_l \frac{m_l r'_l}{M} \right) dr'_1 dr'_2 ... dr'_N dr_C \ . \tag{S1.2.4}$$

Here m_j is the mass of j-th particle, $M = \Sigma m_j$, and the delta-function represents the constraint condition $\Sigma m_l r'_l / M = 0$. Expanding the delta-function into a Fourier integral, we get:

$$\langle 0,0| e^{-iqr_j} |n,k\rangle = A^{-1} \int \Psi_0^* \Psi_n e^{ikr_C} e^{-iqr_C} e^{-iqr'_j} \left[\frac{1}{(2\pi)^D} \int e^{i\frac{m_1}{M} r'_1 p + i\frac{m_2}{M} r'_2 p + ...} dp \right] dr'_1 dr'_2 ... dr'_N dr_C \ . \tag{S1.2.5}$$

Here *D* is the dimensionality of a system, two for the case of excitons in TMDCs. Integration over r_C yields

$$\langle 0,0| e^{-iqr_j} |n,k\rangle = A^{-1} \delta(k-q) \int dp \int \Psi_0^* \Psi_n e^{i\frac{m_1}{M} r'_1 p} e^{i\frac{m_2}{M} r'_2 p} ... e^{i\left(\frac{m_j}{M} p - q\right) r'_j} .... e^{i\frac{m_N}{M} r'_N p} dr'_1 dr'_2 ... dr'_N \ . \tag{S1.2.6}$$

The integral over all "primed" coordinates $r'_1, r'_2, ..., r'_N$ can be expressed in terms of a Fourier transform $\mathcal{F}$ of the product $\Psi_0^* \Psi_n$:

$$\langle 0,0| e^{-iqr_j} |n,k\rangle = A^{-1} \delta(k-q) \int \mathcal{F}\left[\Psi_0^* \Psi_n \right] \left(\frac{m_1}{M} p, \frac{m_2}{M} p, ..., \frac{m_j}{M} p - q, ..., \frac{m_N}{M} p \right) dp \ . \tag{S1.2.7}$$

Let us now plug (S1.2.7) into (S1.2.1). Presence of the delta-function $\delta(k-q)$ in (S1.2.7) reduces summation over three indices $\{n,k,q\}$ in (S1.2.1) to summation over two indices $\{n,q\}$. Then, correction to the EC ground state energy reads:

$$\Delta E_0 = -\frac{1}{2} \frac{1}{A} \sum_S |\rho_{0S}|^2 \frac{2}{\pi} \int_0^\infty \frac{\mathrm{Im} V_s(\omega,k)}{\omega + E_{S0}} d\omega . \tag{S1.2.8}$$

Here summation is performed only over multi-index $S = \{n,k\}$. Due to presence of the delta-function in (S1.2.7) here is no need to explicitly write $\rho_{0S}(k)$, as in (S1.2.1), because momentum k is already included in

the multi-index S. Instead we simply write $\rho_{0S}=\rho_{\{0,0\},\{n,k\}}=\langle 0,0|\rho(k)|n,k\rangle$ rather than $\rho_{0S}(k)$. Since, indices k and q are interchangeable, (S1.2.8) can also be written in terms of the wavenumber q: with potential $V_s(\omega,q)$ and multi-index $S=\{n,q\}$. Such substitution yields equation (2) of the main text.

S1.3. Selection rule for transition charge densities.

Let us now show that in certain cases transition charge densities created by electron and a hole vanish and do not contribute to the energy correction (S1.2.1, S1.2.8). Let us consider linear optical processes, when one absorbed photon creates one electron-hole pair. This photoexcited electron-hole pair can constitute a single neutral exciton or be a part of a larger excitonic complex. The expression for the transition charge density created by this photoexcied pair is written as $\rho_{0S}^{(e-h)}=\rho_{0S}^{h}-\rho_{0S}^{e}$, where ρ_{0S}^{e} is the transition density for the electron and ρ_{0S}^{h} is the transition density for the hole. Using equation (S1.2.7), we get (for $q=k$):

$$\rho_{0S}^{(e-h)}=(2\pi)^{-D}\int dp\left\{\mathscr{F}[\Psi_0^*\Psi_n]\left(...,\frac{m_e}{M}p,...,\frac{m_h}{M}(p-q),...\right)-\mathscr{F}[\Psi_0^*\Psi_n]\left(...,\frac{m_e}{M}(p-q),...,\frac{m_h}{M}p,...\right)\right\}. \tag{S1.3.1}$$

It is easy to see that this expression vanishes if two conditions are met sumultaniously: (i) electron and hole masses are equal: $m_e=m_h=m$ and (ii) the product $\Psi_0^*\Psi_n$ is symmetric with respect to exchange between electron and hole coordinates, i.e. $\{r_e\leftrightarrow r_h\}$: $\Psi_0^*\Psi_n(..,r_e,..,r_h,..)=\Psi_0^*\Psi_n(..,r_h,..,r_e,..)$. If the two conditions are satisfied, then both terms in (S1.3.1) are identical and $\rho_{0S}^{(e-h)}$ vanishes and does not contribute to the sum in (S1.3.2).

Condition (i) can be alternatively expressed in terms of the symmetries of translational part $\exp\left(iq[..+m_er_e+..+m_hr_h+..]/M\right)$ of the total wavefunction $\psi_{q,n}$. If this translational part is symmetric under $\{r_e\leftrightarrow r_h\}$-exchange for any q, this automatically implies that electron and hole masses are equal and condition (i) is satisfied. Condition (ii) can also be expressed in terms of symmetries: it is satisfied when ground and excited state wavefunctions Ψ_0 and Ψ_n are either both symmetric or both antisymmetric with respect to the particle exchange operator $\{r_e\leftrightarrow r_h\}$. Thus, conditions (i) and (ii) can both be expressed in terms of the symmetries of EC wavefunctions: *transition charge densities created by photoexcited electron and hole cancel each other out if both ground- and excited-state wavefunctions are either symmetric or antisymmetric with respect to exchange between electron and hole coordinates*:

$$\{r_e\leftrightarrow r_h\}\begin{pmatrix}\psi_{0,q}\\ \psi_{n,q}\end{pmatrix}=\pm\begin{pmatrix}\psi_{0,q}\\ \psi_{n,q}\end{pmatrix}. \tag{S1.3.2}$$

If at least one of the wavefunctions is asymmetric (i.e. neither symmetric nor antisymmetric), then electron and hole transition charge densities do not cancel each other out and their combined transition charge density does not vanish. Analogously to selection rules in atomic physics, this symmetry-based condition prohibits certain transitions in (S1.2.1, S1.2.8).

S1.4. Charge density matrix element for unbound states.

The summation in (S1.2.8) also runs over bound as well as unbound states (i.e. states corresponding to transition energy above the binding energy $|E_{bind}|$) of an EC. It is important to understand the contribution of

the unbound states to sums in (S1.2.1, S1.2.8). Suppose we have N particles constituting our excitonic complex and one of them (call it particle 1) becomes unbound from the rest. That particle has momentum p in the CM-frame, while the other particles have the total momentum of $-p$. Then the total wavefunction can be written as a product of wavefunction of the first particle

$$\psi_{k,p}^{1,unb.} = A^{-1/2}\exp(ikr_C + ipr_1') \tag{S1.4.1}$$

(superscript "*unb.*" denotes unbound state) and wavefunction of the rest of the particles (remaining EC)

$$\psi_{k,p}^{N-1,unb.} = A^{-1/2}\exp(ikr_C - ipr_C^{N-1})\Psi^{N-1}(r_2', r_3', ...). \tag{S1.4.2}$$

Here A is the sample volume and $r_C^{N-1} = -m_1 r_1' / M_{N-1}$ is the center-of-mass coordinate of the remaining excitonic complex with $M_{N-1} = m_2 + ... + m_N$. CM-wavefunction of the remaining excitonic complex is denoted as Ψ^{N-1}. Thus, the wavefuntion of an unbound EC state reads:

$$\psi_{k,p}^{unb.} = A^{-1}e^{ikr_C}e^{i(1+m_1/M_{N-1})pr_1'}\Psi^{N-1}(r_2', r_3', ...) \equiv A^{-1/2}e^{ikr_C}\Psi_p^{unb.}. \tag{S1.4.3}$$

In order to apply the approach already developed in section S1.2, we brought the wavefunction to the same form as (S1.2.2) by substituting $\Psi_p^{unb.} = A^{-1/2}e^{i(1+m_1/M_{N-1})pr_1'}\Psi^{N-1}(r_2', r_3', ...)$ instead of $\Psi_n(r_1', r_2', ...)$. Note that the total N-particle wavefunction ψ_n has dimensions of $distance^{-DN/2}$ and a CM-wavefunction Ψ_n in (S1.2.2) has a dimensional pre-factor $\sim a_0^{-D(N-1)/2}$, where a_0 is the EC Bohr radius (e.g. in case of 3D hydrogen atom, ground state wavefunction has a dimensional pre-factor $\sim a_0^{-3/2}$). At the same time, according to (S1.4.3), wavefunction $\Psi_p^{unb.}$ has a much smaller pre-factor $A^{-1/2}a_0^{-D(N-2)/2}$. Additionally, the CM-wavefunction (S1.2.2) is localized in space within characteristic length scale proportional to the effective EC Bohr radius a_0. This means that in the momentum space transition charge density, being a Fourier transform of the product of ground- and excited-state wavefunctions, has a characteristic width $\propto 1/a_0$. Thus, a transition charge density (S1.2.7) has maximum at small wavenumbers $q \sim 1/a_0$ and decays at large wavenumbers $q >> 1/a_0$. In the case of transition to an unbound state, due to the factor $\exp[i(1+m_1/M_{N-1})pr_1']$ in (S1.4.3), the maximum of the corresponding transition charge density will be around wavenumber $(1+m_1/M_{N-1})p$ (since presence of the exponent inside a Fourier transform essentially shifts the resulting transformed function). At wavenumbers, significantly different from $(1+m_1/M_{N-1})p$, the transition charge density will go to zero. Thus, at large momentum p, transition charge density $\rho_{0,unb.}(q)$, corresponding to the unbound final state, can be treated as a broadened delta-function $\rho_{0,unb.}(q) \sim \delta(q-[1+m_1/M_{N-1}]p)$. To calculate the contribution of unbound states to (S1.2.8) we plug $\Psi_p^{unb.}$ (as defined in S1.4.3) into equation (S1.2.8). Then, the transition charge density $\rho_{0,unb.}(q)$, acting as a broadened delta-function, selects only high-momentum terms in (S1.2.8):

$$\Delta E_0(n=0 \leftrightarrow unb.) \sim \sum_q \left|\delta\left(q-[1+m_1/M_{N-1}]p\right)\right|^2 \frac{2}{\pi}\int_0^\infty \frac{\mathrm{Im}V_s(\omega,q)}{\omega + E_{S0}}d\omega \sim \tilde{V}_s(E_{S0}, p). \tag{S1.4.4}$$

See section S1.5 for more details regarding calculation of the frequency-integral of the screening potential $\tilde{V}_s$. At high momenta (i.e. at small spatial scales) we see more of a "bare" (i.e. unscreened) charge and hence potential approaches a coulomb-like shape $V \sim 1/p^{D-1} \to 0$.

At the same time, the characteristic magnitude of charge density matrix element $\rho_{0,unb.}$ will be smaller than $\rho_{0,n\sim1}$ (transition charge density for ground to bound state transition) by a factor of $\sim a_0^{D/2}/A^{1/2}$ since, as mentioned above, $\psi_{k,p}^{unb.}$ and $\psi_{k,n\sim1}$ have different dimensional pre-factors.

Let us denote the contributions to (S1.2.8), that correspond to transition to unbound states as $\Delta E_0\left(n=0 \leftrightarrow unb.\right)$. Contributions to ΔE_0, corresponding to transitions to bound states with $n\sim1$ will be denoted as $\Delta E_0\left(n=0 \leftrightarrow bound\right)$. Let us now compare the two contributions:

$$\frac{\Delta E_0\left(n=0 \leftrightarrow unb.\right)}{\Delta E_0\left(n=0 \leftrightarrow bound\right)} \sim \left|\frac{A^{-1/2}}{a_0^{-D/2}}\right|^2 \frac{V_s\left(p >> a_0^{-1}\right)}{V_s\left(p \sim a_0^{-1}\right)} \sim \frac{a_0^D}{A} \frac{1}{V_s\left(p \sim a_0^{-1}\right)} \frac{1}{p^{D-1}}. \quad \text{(S1.4.5)}$$

Let us call (S1.4.5) a "relative contribution of unbound states to the energy correction". Unbound states also have a degeneracy of $g \sim (A^{1/D})^{D-1} p^{D-1}$. Factoring in the degeneracy and assuming that for bound states screening potential is $V_s(p \sim a_0^{-1}) \sim 1/(a_0^{-1})^{D-1}$, equation (S1.4.5) yields

$$g\,\frac{\Delta E_0\left(n=0 \leftrightarrow unb.\right)}{\Delta E_0\left(n=0 \leftrightarrow bound\right)} \sim \frac{a_0}{A^{1/D}} \quad \text{(S1.4.6).}$$

Expression (S1.4.6) evaluates relative contribution of all states with momentum absolute value lying between $|p|$ and $|p| + A^{-1/D}$ (here $A^{-1/D}$ plays the role of an elementary momentum). Let us now re-write this expression per unit energy – i.e., relative contribution of unbound states with energies between $M|p|^2/2$ and $M(|p| + A^{-1/D})^2/2$:

$$\frac{g\Delta E_0\left(n=0 \leftrightarrow unb.\right)}{\Delta E_0\left(n=0 \leftrightarrow bound\right)} / dE_{S0} \sim \frac{1}{M|p|A^{-1/D}} \frac{a_0}{A^{1/D}} \propto \frac{1}{\sqrt{E_{S0}}}. \quad \text{(S1.4.7)}$$

Here $dE_{S0} \sim M|p|A^{-1/D}$ plays the role of elementary energy. Thus, contribution of high-energy unbound states to (S1.2.1, S1.2.8) indeed vanishes for transition energies E_{S0} much higher than the binding energy $|E_{bind.}|$.

S1.5. Integral of imaginary part of potential. Its physical significance and properties.

In this section we derive equation (3) of the main text. This equation helps to further simplify equations (S1.2.1, S1.2.8). Below we show that integral of the imaginary part of the screening potential $\tilde{V}_s = 2\pi^{-1}\int_0^\infty \mathrm{Im}V_s\left(\omega,q\right)/\left(\omega+E\right)d\omega$, entering (S1.2.1, S1.2.8), can be expressed in terms of the real part of the screening potential which can be calculated using simple Poisson equation. We use Kramers-Kronig relations to calculate the imaginary part $\mathrm{Im}V_s\left(\omega,q\right)$ from the real function $V_s'(\omega,q) = \mathrm{Re}V_s\left(\omega,q\right)$:

$$\tilde{V}_s(E)=\frac{2}{\pi}\int_0^\infty d\omega\frac{1}{\pi}\int_{-\infty}^{\infty}d\omega'\frac{1}{\omega+E}\frac{-1}{\omega-\omega'}V_s(\omega')=-\frac{2}{\pi^2}\int_{-\infty}^{\infty}d\omega\frac{1}{\omega+E}\ln\left|\frac{\omega}{E}\right|V_s'(\omega).$$

(S1.5.1)

Equation 1.5.1 can be viewed as a linear transformation of $V_s(\omega)$. Let's denote this transformation as $\mathscr{T}$:

$$\tilde{V}_s(E)\equiv\mathscr{T}\left[V_s'(\omega)\right](E). \tag{S1.5.2}$$

Let us analyze properties of transformation $\mathscr{T}$. Since potential is a real-valued function in time domain, it is symmetric in the frequency domain: $V_s(\omega)=V_s^*(-\omega)$. Using this symmetry, we re-write (S1.5.1) as

$$\tilde{V}_s=-\frac{2}{\pi^2}\int_0^\infty d\omega\frac{1}{\omega+E}\ln\left|\frac{\omega}{E}\right|V_s'(\omega)-\frac{2}{\pi^2}\int_0^\infty d\omega\frac{1}{-\omega+E}\ln\left|\frac{\omega}{E}\right|V_s'(\omega)=-\frac{4}{\pi^2}\int_0^\infty d\omega\frac{E}{E^2-\omega^2}\ln\left|\frac{\omega}{E}\right|V_s'(\omega).$$

(S1.5.3)

Introducing notation $\omega_{Log}=\ln\omega/\Omega$ (logarithmic frequency) and $V_s'^{Log}\left(\omega_{Log}\right)=V_s'\left(\Omega e^{\omega_{Log}}\right)$ (here Ω is an arbitrary unit frequency, for example, we can choose Ω =1Hz), the integral $\tilde{V}_s$ becomes:

$$\tilde{V}_s=\frac{4}{\pi^2}\int_{-\infty}^{\infty}\frac{E_{Log}-\omega_{Log}}{e^{E_{Log}-\omega_{Log}}-e^{-\left(E_{Log}-\omega_{Log}\right)}}V_s'^{Log}\left(\omega_{Log}\right)d\omega_{Log}\equiv\left(\frac{2}{\pi^2}\frac{E_{Log}}{\sinh E_{Log}}\right)*V_s'^{Log}\left(E_{Log}\right). \tag{S1.5.4}$$

Here symbol "$*$" denotes convolution. Thus, in a logarithmic scale, $\tilde{V}_s$ in equation (2) of the main text is nothing but frequency-dependent potential smoothened by a normalized bell-shaped function $2\pi^{-2}x/\sinh x$. Average value of such function is 0 and its standard deviation is ~2.2.

S1.6. Approximation for dynamic screening.

Let us now further simplify (S1.2.8) and derive equation (4) of the main text. First, we re-write (S1.2.1) truncating transitions that do not contribute to it (symmetric transitions and transitions to high-energy unbound states):

$$\Delta E_0\approx-\frac{1}{2A}\sum_q\sum_{S=S_{\min}}^{S_{\max}}\left|\rho_{0S}(q)\right|^2\tilde{V}_s\left(E_{S0},q\right). \tag{S1.6.1}$$

Here $S_{\min}$ is lowest asymmetric state and $S_{\max}$ - unbound state with corresponding transition energy of $\sim|E_{bind.}|$. As mentioned in the main text, this equation with frequency-dependent interaction potential can be re-written by replacing the transition energy E_{S0} by an effective constant energy E_{eff}, lying between minimum and maximum bounds $E_{\min}$ and $E_{\max}$. Such substitution can be done based on the second mean value theorem for integrals: $\int_a^b f(\omega)g(\omega)d\omega=f(c\in[a,b])\int_a^b g(\omega)dx$. Thus, we can pull the frequency-dependent $\tilde{V}_s$ out of the summation over S:

$$\Delta E_0 \approx -\frac{1}{2A}\sum_q \tilde{V}_s\left(E_{eff},q\right)\sum_{S=S_{min}}^{S_{max}}\left|\rho_{0S}\left(q\right)\right|^2 \qquad \text{(S1.6.2)}$$

with $E_{min} < E_{eff} < E_{max}$.

Using definition of transition charge density (S1.2.3) and completeness of the set of state-vectors $\left|S\right\rangle$, we rewrite (S1.6.2) as:

$$\Delta E_0 = -\frac{1}{2A}\sum_q\left\{\sum_S\left\langle 0\right|\rho\left(q\right)\left|S\right\rangle\left\langle S\right|\rho\left(q\right)\left|0\right\rangle\tilde{V}_s\left(E_{eff},q\right)\right\} = \left\langle 0\right| -\frac{1}{2A}\sum_q\rho^2\left(q\right)\tilde{V}_s\left(E_{eff},q\right)\left|0\right\rangle. \qquad \text{(S1.6.3)}$$

Equation (S1.6.3) is formally identical to the energy correction in the first-order perturbation theory. The expression between $\left\langle 0\right|$ and $\left|0\right\rangle$ in (S1.6.3) is nothing but the energy of a field created by charge density ρ - i.e. interaction and self-action potential energies of particles constituting the EC. This energy can be also expressed as $\tilde{U}_s = \sum_{j,k}^{N} Q_j Q_k \tilde{V}_s\left(E_{eff}, r_j - r_k\right)$. Adding (S1.6.3) to the unperturbed EC energy we get the perturbed EC ground state energy:

$$E_0' = E_0 + \Delta E_0 = \left\langle 0\right|T + U_0\left|0\right\rangle + \left\langle 0\right|\tilde{U}_s\left(E_{eff}\right)\left|0\right\rangle = \left\langle 0\right|T + \tilde{U}\left(E_{eff}\right)\left|0\right\rangle, \qquad \text{(S1.6.4)}$$

where T and U_0 are total kinetic and potential energies of unperturbed EC. Expression (S1.6.4) simply suggests that in order to evaluate binding energy of a dynamically screened EC we just need to solve a Schrodinger equation with potentials calculated with medium dielectric functions taken at energy E_{eff}.

S2. Modelling of ECs in 2D systems.

S2.1. Analysis of interaction potential.

In our experimental study, monolayer TMDC lies on top of the SiO_2 substrate (dielectric constant ~3), while environment on top of TMDC is varied. Interaction potential between two charges inside such a thin dielectric layer was derived by L. Keldysh[3]:

$$V(\rho)=\frac{\pi}{\varepsilon d}\left[H_0\left(\frac{\varepsilon_{top}+\varepsilon_{bot}}{\varepsilon d}\rho\right)-Y_0\left(\frac{\varepsilon_{top}+\varepsilon_{bot}}{\varepsilon d}\rho\right)\right]. \qquad \text{(S2.1.1)}$$

Here ρ is spatial separation between the particles, ε and d are dielectric constant and thickness of 2D material, $\varepsilon_{top},\varepsilon_{bot}$ are dielectric constants of top and bottom (substrate) environments; H_0,Y_0 are Struve function and Bessel function of the second kind respectively. This equation was derived with assumption of d much smaller than the separation between the charged particles, and of $\varepsilon >> \varepsilon_{top},\varepsilon_{bot}$. These assumptions hold in some of our experimental systems, e.g. for a WS_2 monolayer ($\varepsilon \sim 10$) stacked between SiO_2 substrate ($\varepsilon_{bot} \sim 3$) and either vacuum ($\varepsilon_{top}=1$) or ionic liquid ($\varepsilon_{top} \sim 2$ for frequencies higher than GHz). Unfortunately, for other systems these assumptions fail. Therefore, in this section we re-derive screened potentials for the geometries relevant in our experiments.

Heterostructure of two thin semiconductors.

Since equation (S2.1.1) was derived with the assumption of homogeneous dielectric constant of the thin-layer material, it cannot be directly applied to stacked heterostructures of two different 2D materials (e.g. WS_2/MoS_2, WS_2/graphene). In case of a homogeneous monolayer material the potential $V(\rho)$ can be obtained as a solution of Poisson's equation

$$\nabla^2 V=-\delta(\vec{r})+\chi_{2D}\delta_d(z)\nabla^2_{\vec{\rho}}V \quad , \qquad \text{(S2.1.2)}$$

where $\vec{\rho}$ is in-plane coordinate, z - out-of-plane coordinate, $\vec{r}=(\vec{\rho},z)^T$, and $\chi_{2D}=(\varepsilon-1)d \approx \varepsilon d$ is a 2D analogue of polarizability. Note that in equation (S2.1.1) ε and d enter only as a product $\varepsilon d \approx \chi_{2D}$. The term $\delta(\vec{r})$ represents charge density creating the potential (we set elementary charge to 1) and broadened delta-function $\delta_d(z)$ indicates that our 2D crystal is localized in the x-y plane and has small thickness d. Cudazzo, Tokatly and Rubio[4] elegantly show that the solution of (S2.1.2) has exactly the same form as (S2.1.1). The 2D polarization density $\chi_{2D}=\chi_{3D}d$ characterizes dipole moment induced by electric field per unit area. In the case of a heterostructure of two monolayer materials, the 2D polarizabilities of two thin materials ($\chi_{2D}^{(1)}$ and $\chi_{2D}^{(2)}$) are additive. Thus, under the effective medium approximation, our heterostructure can be formally treated as a single (homogenious) material with effective 2D polarizability

$$\chi_{2D}^{eff}=\chi_{2D}^{(1)}+\chi_{2D}^{(2)}. \qquad \text{(S2.1.3)}$$

This means that dealing with heterostructures (SiO_2/WS_2/graphene, $SiO_2/WS_2/MoS_2$, $SiO_2/WS_2/WS_2$), we can still use Keldysh equation (S2.1.1). In that equation εd must be substituted by $\chi_{2D}^{eff} \approx \varepsilon d+(\varepsilon'-1)d'$, where ε' and d' are the dielectric constant and the thickness of the screening 2D material deposited on top of our WS_2.

Monolayer semiconductor between environments $\varepsilon \approx \varepsilon_{bot} \approx \varepsilon_{top}$.

Now let us consider how a monolayer semiconductor is screened by different 3D environments. We start with the case when surrounding environments have dielectric constants similar to the dielectric constant of our 2D material. In this case, interaction potential is simply reduced to the Coulomb form:

$$V(\rho) = \frac{1}{\varepsilon\rho}. \quad \text{(S2.1.4)}$$

Interestingly, for small thickness d, argument of Struve and Bessel functions in (S2.1.1) is always large ($\rho / d >> 1$) and in this limit, $H_0(x>>1) - Y_0(x>>1) \approx 2\pi^{-1}x^{-1}$. In this case, (S2.1.1) also yields a Coulomb potential $1/\varepsilon\rho$. Thus, equation (S2.1.1), derived under more strict assumptions, is still valid for the case of $\varepsilon \approx \varepsilon_{bot} \approx \varepsilon_{top}$.

Monolayer semiconductor between environments $\varepsilon_{bot} << \varepsilon \approx \varepsilon_{top}$.

Condition of $\varepsilon_{bot} << \varepsilon \approx \varepsilon_{top}$ occurs, for example, when WS_2 is deposited on a SiO_2 substrate and is covered by a strongly screening 3D medium, such as ionic liquid. In this case, equation (S2.1.1) needs to be rederived. In momentum space, the potential $V(k)$ for any thickness and any set of dielectric constants reads[3]:

$$V(k) = \frac{2\pi}{\varepsilon|k|}\frac{1+(A_1+A_2)e^{-|k|d} + A_1A_2e^{-2|k|d}}{1-A_1A_2e^{-2|k|d}} = \frac{4\pi}{\varepsilon|k|}\frac{\cosh\left(|k|\frac{d}{2}+\eta_2\right)\cosh\left(|k|\frac{d}{2}+\eta_1\right)}{\sinh\left(|k|d+\eta_1+\eta_2\right)}. \quad \text{(S2.1.5)}$$

Here $A_{1,2} = (\varepsilon - \varepsilon_{bot,top})/(\varepsilon + \varepsilon_{bot,top})$ and $\eta_{1,2} = -(1/2)\ln A_{1,2}$. Substituting $\varepsilon_{bot} << \varepsilon \approx \varepsilon_{top}$ we get $A_2 << A_1 \sim 1$ and hence,

$$V(\rho) \approx \int \frac{d^2k}{(2\pi)^2}e^{ik\rho}\frac{2\pi}{\varepsilon|k|}\frac{1+A_1e^{-|k|d}+\mathrm{O}(A_2)}{1-\mathrm{O}(A_2)} \approx \frac{1+A_1}{\varepsilon\rho} \approx \frac{1}{\frac{\varepsilon_{top}+\varepsilon_{bot}}{2}\rho}. \quad \text{(S2.1.6)}$$

Like in the previous case, for small d, Coulomb-like potential (S2.1.6) can be approximated as

$$V(\rho) \approx \frac{1}{\frac{\varepsilon_{top}+\varepsilon_{bot}}{2}\rho} \approx \frac{\pi}{\varepsilon d}\left[H_0\left(\frac{\varepsilon_{top}+\varepsilon_{bot}}{\varepsilon d}\rho\right) - Y_0\left(\frac{\varepsilon_{top}+\varepsilon_{bot}}{\varepsilon d}\rho\right)\right]. \quad \text{(S2.1.7)}$$

Strongly screening top environment ($|\varepsilon_{top}| >> \varepsilon$*).*

When dealing with a WS_2/graphene heterostructure in the low-frequency regime, graphene can be treated as an ideal conductor. In this case $A_2 \approx -1$ and

$$V(\rho) = \int \frac{d^2k}{(2\pi)^2}e^{ik\rho}\frac{2\pi}{\varepsilon|k|}\frac{1+(A_1-1)e^{-|k|d} - A_1e^{-2|k|d}}{1+A_1e^{-2|k|d}}. \quad \text{(S2.1.8)}$$

We can analyze this expression in two limit cases:

$$V\left(\rho << d\right) \approx \frac{1}{\varepsilon\rho} \text{ and } V\left(\rho >> d\right) \approx \frac{1}{\varepsilon\rho} - \frac{1}{\varepsilon\sqrt{\rho^2 + \left(\frac{1+3A_1}{1+A_1}\right)d^2}} \quad \text{(S2.1.9)}$$

This limit behavior is identical to the potential of a vertical dipole of the size $\sqrt{(1+3A_1)/(1+A_1)}d$.

For small TMDC thickness, field created by such dipole will be vanishingly small and can be written as equation (S2.1.1) putting top dielectric constant to infinity.

Summary.

Surprisingly, the Keldysh equation (S2.1.1) can be used for all experimentally relevant cases $\varepsilon >> \varepsilon_{bot}, \varepsilon_{top}; \varepsilon_{bot} << \varepsilon \approx \varepsilon_{top}; \varepsilon_{bot} \approx \varepsilon \approx \varepsilon_{top}; \varepsilon_{bot} << \varepsilon << \varepsilon_{top}, \varepsilon_{bot} \sim \varepsilon << \left|\varepsilon_{top}\right|$. For heterostructures, 2D polarizability $\varepsilon d + \left(\varepsilon' - 1\right) d'$ should be substituted into (S2.1.1) instead of εd (ε, d, ε', d' are dielectric constants and thicknesses of materials in the heterostructure).

S2.2. Numerical calculations.

Having justified the applicability of equation (S2.1.1) to the systems studied in this work, we performed numerical calculations of the binding energies of ECs bound by the Keldysh interaction potential.

Binding energies were calculated by variationally solving the *N*-body Schrodinger equation with the Hamiltonian defined in equation (S1.6.4) or, equivalently, equation (4) of the main text. The potential in that equation was taken in the form (S2.1.1). We used electron and hole masses of 0.45 electron mass[5,6] and WS_2 thickness of 0.7nm. The trial wavefunction was defined as a linear combination of correlated Gaussian basis functions. The variational parameters of the Gaussians were chosen via random trial and error, a process known as the stochastic variational method[7,8]. A finite number of parameter sets is generated, and the one which yields the lowest total energy is used to define a new correlated Gaussian which is added to the basis set. We used 40 basis functions to model neutral excitons and 400 functions to model trions and defect-bound excitons.

We calculated upper and lower bounds of EC binding energies in the following way. We evaluated the dielectric function of WS_2, top environment, and the bottom environments at two frequencies, $\omega_{\min}$ and $\omega_{\max}$, prescribed by our model. These effective dielectric constants were inserted into the Keldysh potential (S2.1.1) entering the Hamiltonian (S1.6.4). The two values of EC binding energy obtained by variational minimization of that Hamiltonian serve as upper and lower bounds for the estimated EC binding energy. For neutral exciton, the minimum frequency ($\omega_{\min}$) is 130meV (transition energy between ground and first excited states) and the maximum frequency ($\omega_{\max}$) is 320meV (neutral exciton binding energy). For trion, the minimum frequency is 1meV (limited by lifetime) and the maximum frequency is 30meV (characteristic trion binding energy). For defect-bound exciton, we assume 0meV effective frequency. The effective dielectric constants evaluated using this approach are shown in Table S2.2.1. The exciton binding energies calculated using these effective dielectric constants are shown in Fig.2 of the main text.

Device structure \ EC type		Neutral exciton at ω_{min}	Neutral exciton at ω_{max}	Trion at ω_{min}	Trion at ω_{max}	Defect-bound exciton at ω_{min}
SiO_2/WS_2	$\varepsilon(\omega)$	14	16	5	16	5
	$\varepsilon_{top}(\omega)+\varepsilon_{bot}(\omega)$	3	3	5	3	5
SiO_2/WS_2/graphene	$\varepsilon(\omega)$	16*	18*	5	18*	5
	$\varepsilon_{top}(\omega)+\varepsilon_{bot}(\omega)$	3	3	∞	3	∞
SiO_2/WS_2/ionic liquid	$\varepsilon(\omega)$	14	16	5	16	5
	$\varepsilon_{top}(\omega)+\varepsilon_{bot}(\omega)$	4	4	7	4	21
$SiO_2/WS_2/MoS_2$	$\varepsilon(\omega)$	28*	32*	10*	32*	10*
	$\varepsilon_{top}(\omega)+\varepsilon_{bot}(\omega)$	3	3	5	3	5

Table S2.2.1. Effective values of dielectric functions of our 2D material (WS_2), and surrounding environments. The top number is the value of $\varepsilon(\omega)$ - the dielectric constant of the intermediate layer (WS_2 or heterostructure) – taken at minimum and maximum frequencies. The bottom number is the combined dielectric constant of 3D materials surrounding the thin layer $\varepsilon_{top}(\omega)+\varepsilon_{bot}(\omega)$ also taken at minimum and maximum frequencies. The symbol (*) indicates that the device was modelled as a heterostructure with effective dielectric constant ε of the intermediate layer replaced by the sum of WS_2 dielectric constant and dielectric constant of another monolayer material deposited on top (MoS_2 or graphene at high frequencies).

To test our computational methods and parameters we compared results of our modeling to the result obtained by other researchers using quantum Monte-Carlo calculations (Table S2.2.2). We do see a good qualitative and quantitative agreement between our values.

Exciton type	Our calculations	From Ganchev *et al.*[47]	From Szyniszewski *et al.*[46]
1L WS_2: Trion	18meV – 50meV	30meV	31meV
1LWS_2: Defect-bound exciton	180meV	150meV	
2L WS_2: Trion	13meV – 20meV	15meV	16meV
2LWS_2: Defect-bound exciton	100meV	75meV	

Table S2.2.2. Binding energies of trions and defect-bound excitons in 1L and 2L WS_2 obtained via different methods.

S3. Artifact analysis.

Multiple effects other than dynamic screening may, potentially, affect the EC peak positions. Some of these effects are chemical modification of the samples, screening by free carriers, and effects of strain. In this section, we analyze these effects and show they cannot account for the shifts seen in Fig.2 of the main text.

S3.1. WS_2 devices in liquids. Chemical modification.

We tested possible contribution of chemical reactions between WS_2 and its environment to the data displayed in Fig.2 of the main text. While WS_2 is not known to enter chemical reactions in our conditions (vacuum, low temperatures, gate voltages ~1V)[9], such reactions may potentially affect the position of defect-related exciton peak for devices in ionic liquids. We monitored the position of the defect-related peak while first depositing the liquid onto WS_2, thermally cycling the device between 78K and 340K, and finally removing the liquid. We observed that the peak returns to its original position in the end of the cycle (Fig.S3.1.1). This suggests that ionic liquids do not induce significant permanent chemical changes in WS_2 surface.

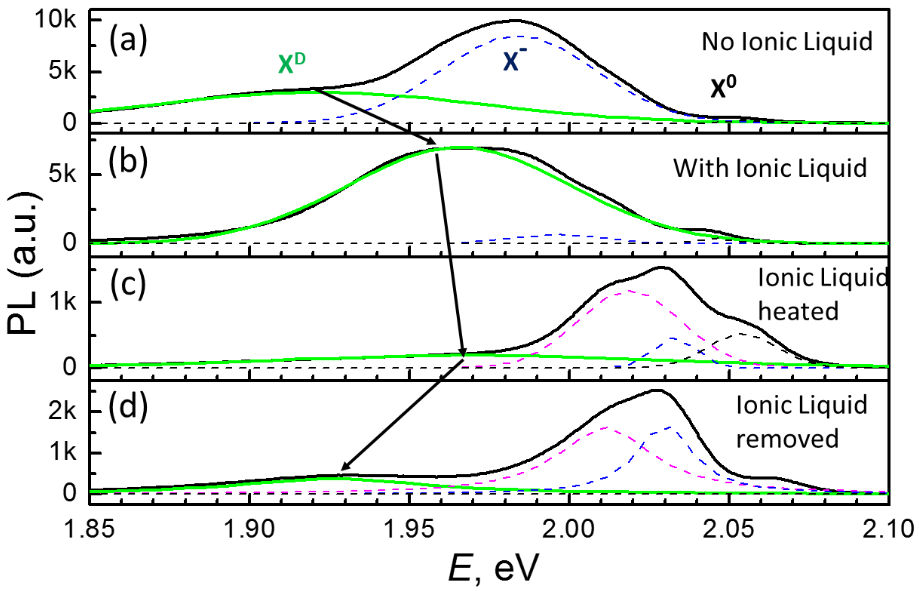

Figure S3.1.1. Evolution of WS_2 photoluminescence spectra during deposition and removal of ionic liquid. All measurements were performed at 78K temperature. **(a)** PL spectrum for a pristine WS_2 flake in vacuum. **(b)** PL spectrum recorded after warming the sample up to room temperature, drop-casting of ionic liquid onto it, and subsequent cooling down to 78K. **(c)** PL spectrum acquired after heating the sample to 340K, keeping it at 340K for 3 hours, and cooling it down to 78K. **(d)** PL spectrum recorded at 78K after the removal of the ionic liquid. Sample was again heated up to room temperature, ionic liquid was removed by rinsing the device with isopropanol. After that the sample was again cooled down.

Additionally, we performed experiments in which a different type of liquid – water – was deposited onto WS_2 samples under ambient conditions. We observed that the presence of water affects neutral and defect-bound exciton peaks in a similar way as ionic liquid (Fig. S3.1.2). Independence of the observed effects on chemical composition of the liquid confirms that peak shifts are not caused by chemical factors.

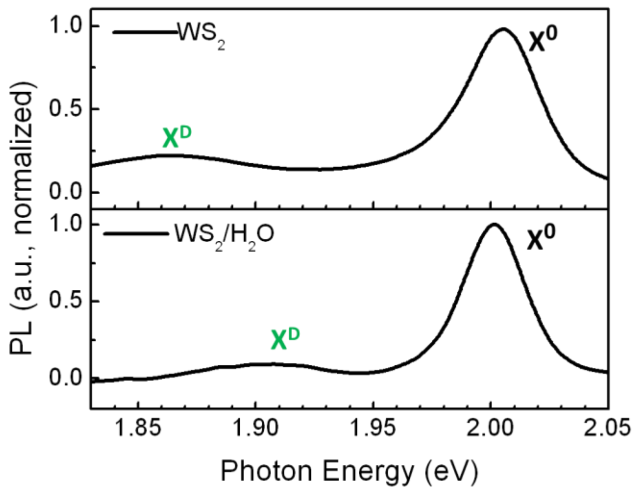

Figure S3.1.2. (a) Photoluminescence spectrum of a pristine SiO_2/WS_2 device in ambient conditions. (**b)** Photoluminescence spectrum of the $SiO_2/WS_2/H_2O$ device. The presence of water causes a ~40meV blue-shift of the defect-bound exciton peak compared to the device in air. Neutral exciton does not shift, within 3meV precision.

S3.2. Screening by free carriers.

Another mechanism of screening of electric interactions is screening by free carriers. Free-carrier screening causes dielectric functions to become wavenumber-dependent. In our measurements, we deal with two types of screening with different characteristic spatial scales:

(a) In WS_2/graphene heterostructures, the fields of ECs in WS_2 are screened by relativistic carriers in graphene. Due to absence of the bandgap and linear dispersion charge carriers, the only characteristic spatial scale in graphene is its lattice constant *a*~0.3nm. Then, the Thomas-Fermi screening length in graphene is also ~0.3nm[10]. At smaller spatial scales (larger momenta) screening is akin to weak screening by individual atoms and effective dielectric constant is $\varepsilon(q<1/a)\sim 1$, while screening becomes stronger at momenta smaller than $1/a$. Hence, for ECs in WS_2 with the effective Bohr radii 1~2nm graphene can be treated as a strongly screening metal, at least in the regime of low frequencies[10,11].

(b) The electric fields of ECs in WS_2 can also be screened by free carriers in WS_2 itself. Presence of free carriers in the material is typically caused by doping (intrinsic or induced). To investigate the effects of free-carrier screening we fabricated back-gated WS_2 samples on Si/SiO_2 substrate (with 300nm SiO_2 thickness) with controllable doping level. Experimentally, our data (Fig.S3.2.1) as well as the data obtained by other groups[12,13] suggest that ECs are relatively weakly affected by the presence of free carriers. Neutral exciton peak shifts from 2.055eV in a depleted sample to 2.075eV in a doped sample (Fig.S3.2.1). The position of the defect-bound exciton peak shifts by <5meV due to gating (Fig.S3.2.1). In depleted samples the binding energy of trions is the smallest, 24meV. The trion peak experiences red-shifts in positively gated devices. This

reflects the increase of the trion binding energy in presence of doping. Peak shifts observed in our experiments, described in the main text, exceed shifts that can be induced by doping: Neutral exciton red-shift to ~2.045eV in bilayer WS_2 samples (Fig.3.3.1) and in WS_2/MoS_2 heterostructures. Defect-related peaks blue-shift by ~40meV in presence of graphene or liquid environments. Trion binding energy becomes as low as 19meV in presence of graphene. Thus, screening by electron gas in 2D semiconductor cannot account for peak shifts observed in our experiments.

Relatively weak effects of free-carrier screening are also expected theoretically. In the case of screening by gas of massive 2D electrons, effective dielectric function depends on the wavenumber or, in other words, on the spatial scale at which interactions occur: dielectric function becomes large only at interparticle distances greater than characteristic screening length. The screening length lies between the exciton Bohr radius $a_0 > 1nm$ and inverse Fermi momentum k_F^{-1}[14]. For our typical $10^{12}cm^{-2}$ doping level, inverse Fermi momentum is $k_F^{-1} \sim 5nm > a_0$. In this regime the material dielectric function is[14]:

$$\varepsilon(q) = \varepsilon_0 \left(1 + \frac{g/a_0}{q} \left\{ 1 - \sqrt{1 - \left[\frac{2k_F}{q} \right]^2} \right\} \right) \approx \varepsilon_0 \left(1 + \frac{g/a_0}{q} \frac{2k_F^2}{q^2} \right).$$

Here ε_0 is the dielectric constant at large momenta (small spatial scales), $g = 4$ is a spin- and valley- degeneracy factor and q - wavenumber. Thus, in the case of a *moderate doping* ($10^{12}cm^{-2}$) at small spatial scales corresponding to exciton size (1~2nm), the 2D material dielectric function is close to ε_0 (dielectric function in absence of free carriers).

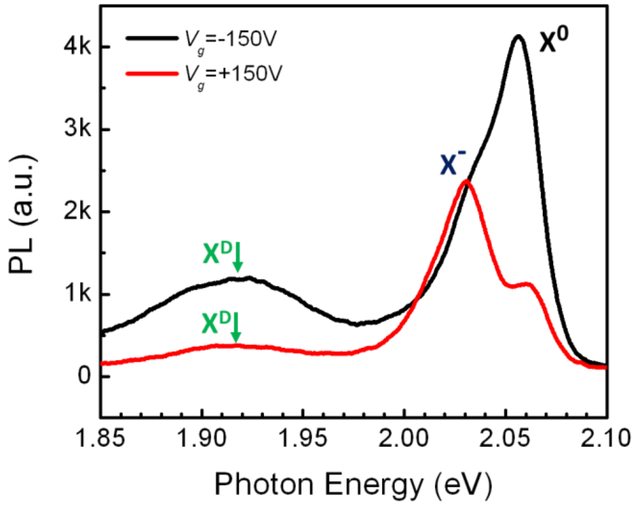

Figure S3.2.1. Photoluminescence spectra of a gated WS_2 device acquired at two different gate voltages, V_g=-150V and V_g=+150V.

S3.3. Effects of strain.

Mechanical strain may be induced in WS_2 during transfer of another material onto it. In principle, strain may affect EC peak positions[15]. To roughly estimate the magnitude of that strain, we compared PL spectra of WS_2 in a mechanically transferred WS_2/MoS_2 heterostructure and of natural WS_2 bilayer which did not undergo any transfer (Fig.S.3.3.1). While screening in these structures is very similar, the transfer-related strain may only be present in WS_2/MoS_2 device. The comparison of X^0 peak positions between the spectra of WS_2/MoS_2 heterostructure and natural bilayer WS_2 shows only ~2meV difference and suggests that the stain imparted by the transfer is smaller than ~0.03%[15]. This strain and corresponding peak shift is too small to account for the trends seen in Fig.2 of the main text.

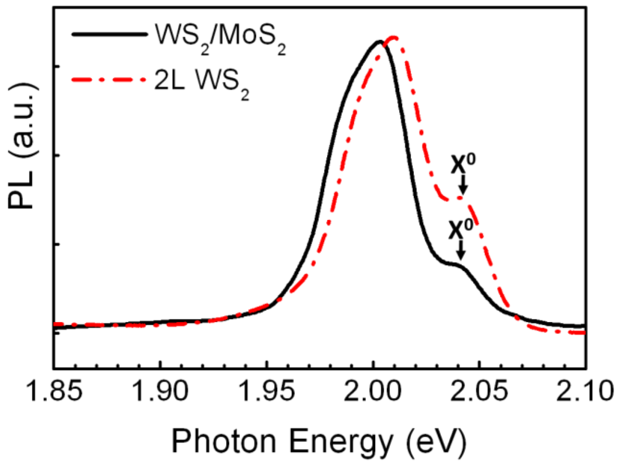

Figure 3.3.1. Photoluminescence spectra of artificial WS_2/MoS_2 heterostructure and natural bilayer WS_2.

[1] R. Zimmermann, K. Kilimann, W. Kraeft, D. Kremp, and G. Röpke, physica status solidi (b) **90**, 175 (1978).

[2] H. Haug and S. Schmitt-Rink, Progress in Quantum Electronics **9**, 3 (1984).

[3] L. V. Keldysh, Pisma Zh. Eksp. Teor. Fiz **29**, 658 (1979).

[4] P. Cudazzo, I. V. Tokatly, and A. Rubio, Physical Review B **84**, 085406 (2011).

[5] A. Ramasubramaniam, Physical Review B **86**, 115409 (2012).

[6] M. Dendzik *et al.*, Physical Review B **92**, 245442 (2015).

[7] J. Mitroy *et al.*, Reviews of Modern Physics **85**, 693 (2013).

[8] K. Varga, Computer Physics Communications **179**, 591 (2008).

[9] Y. Zhang, J. Ye, Y. Matsuhashi, and Y. Iwasa, Nano letters **12**, 1136 (2012).

[10] M. Katsnelson, Physical Review B **74**, 201401 (2006).

[11] V. Despoja, D. Mowbray, D. Vlahović, and L. Marušić, Physical Review B **86**, 195429 (2012).

[12] A. Chernikov, A. M. van der Zande, H. M. Hill, A. F. Rigosi, A. Velauthapillai, J. Hone, and T. F. Heinz, Physical review letters **115**, 126802 (2015).

[13] B. Zhu, X. Chen, and X. Cui, Scientific Reports **5**, 9218 (2015).

[14] T. Ando, A. B. Fowler, and F. Stern, Reviews of Modern Physics **54**, 437 (1982).

[15] H. J. Conley, B. Wang, J. I. Ziegler, R. F. Haglund Jr, S. T. Pantelides, and K. I. Bolotin, Nano letters **13**, 3626 (2013).
